# Pixel Intensity Resemblance Measurement and Deep Learning Based Computer Vision Model for Crack Detection and Analysis

**DOI:** 10.3390/s23062954

**Published:** 2023-03-08

**Authors:** Nirmala Paramanandham, Kishore Rajendiran, Florence Gnana Poovathy J, Yeshwant Santhanakrishnan Premanand, Sanjeeve Raveenthiran Mallichetty, Pramod Kumar

**Affiliations:** 1 School of Electronics Engineering, Vellore Institute of Technology, Chennai Campus, Chennai 600127, India; 2 Department of ECE, Sri Sivasubramaniya Nadar College of Engineering, Kalavakkam 603110, India; 3 Department of Software Engineering, Rochester Institute of Technology, GoLisano College of Computing and Information Sciences, Rochester, NY 14623, USA; 4 Department of Mechanical and Industrial Engineering, Northeastern University, Boston, MA 02115, USA

**Keywords:** cracks, deep learning, detection, images, noise, integrity, safety

## Abstract

This research article is aimed at improving the efficiency of a computer vision system that uses image processing for detecting cracks. Images are prone to noise when captured using drones or under various lighting conditions. To analyze this, the images were gathered under various conditions. To address the noise issue and to classify the cracks based on the severity level, a novel technique is proposed using a pixel-intensity resemblance measurement (PIRM) rule. Using PIRM, the noisy images and noiseless images were classified. Then, the noise was filtered using a median filter. The cracks were detected using VGG-16, ResNet-50 and InceptionResNet-V2 models. Once the crack was detected, the images were then segregated using a crack risk-analysis algorithm. Based on the severity level of the crack, an alert can be given to the authorized person to take the necessary action to avoid major accidents. The proposed technique achieved a 6% improvement without PIRM and a 10% improvement with the PIRM rule for the VGG-16 model. Similarly, it showed 3 and 10% for ResNet-50, 2 and 3% for Inception ResNet and a 9 and 10% increment for the Xception model. When the images were corrupted from a single noise alone, 95.6% accuracy was achieved using the ResNet-50 model for Gaussian noise, 99.65% accuracy was achieved through Inception ResNet-v2 for Poisson noise, and 99.95% accuracy was achieved by the Xception model for speckle noise.

## 1. Introduction

The identification of cracks on different types of structures has always been tedious and time consuming work. Regular checks have to be made in order to prevent any serious damage to infrastructure. Traditional inspections would require the use of specialized personnel to manually check for any cracks. This process is greatly complicated when it has to be done in areas such as roads, bridges and highways. It can cause disturbance to regular work or create traffic due to the need to employ additional platforms or machinery for aiding in the inspection process. Furthermore, after the examination, the reports are usually checked manually to identify the underlying issues. This procedure is time consuming and costly to implement. In order to reduce the cost, time and labor involved in such scenarios, the use of unmanned aerial vehicles (UAV) and transfer learning methods can be used to identify cracks. Once a crack is identified, it can be separated, based on the severity level. This tedious process would be simplified by automation. The main goal of this research article is to improve this nondestructive method of investigation which can be employed at a considerably lower cost, while maintaining good accuracy. The convolutional neural network (CNN) method is one of the most efficient network methods which can be used for image-based crack detection. CNNs have demonstrated much higher accuracy than many image-enhancement algorithms, such as the morphological approach, edge detectors, wavelet analysis, etc. [1].

Over the years, machine learning and convolutional networks have been displaying strong capability for feature extraction and target detection and many researchers have developed and studied various applications for improving the accuracy. Research carried out by Cuong Nguyen Kim et al. [1] concentrated on pre-processing. To improve the accuracy, Aravinda S Rao et al. [2] took a different approach in pre-processing where they divided the image into multiple patches before the images were fed into the CNN-based algorithms. Vishal Mandal et al. [3] focused on detecting the cracks. Similar methods have been implemented in various research, such as in Rupinder Pal Singh et al.’s [4] work, where they used a bilateral filter to remove noise as it helped to preserve edges while reducing noise. Another work carried out by Mukund N Naragund et al. [5] showcased a methodology using wavelet transform along with a bilateral filter to denoise and preserve detail to a high degree.

The main contributions of this research are summarized as follows:

1. A deep learning model for crack detection using image processing for computer vision is proposed;
2. In order to detect whether the image has been affected by noise, a unique technique which uses pixel-intensity resemblance is implemented;
3. A binarization-skeletonization-edge detection (BSE) algorithm is proposed for estimating the width of cracks. Based on the width, the images are segregated into high-risk, medium-risk and low-risk cracks using preset thresholds.

Section 2 elaborates the available literature on crack detection. Section 3 explains the proposed work. Section 4 and Section 5 discuss the results and conclusions.

## 2. Background

Cracks can be detected using basic machine learning algorithms. As opting for deep learning is more fruitful in terms of accuracy and speed, many state-of-the-art techniques have concentrated in these techniques. The research article by Raza Ali et al. [6] surveyed different CNN-based algorithms and stated that Unet was the best performer when compared to Pixelnet, Alexnet, Googlenet and a few other algorithms. V Mandal et al. [3] was able to detect cracks in real time by mounting a camera on the dashboard of a moving car. Y Zhang et al. [7] used the YOLO v3 algorithm as a base and was able to detect the cracks efficiently by using MobileNet for transfer learning and the convolutional block attention model. The authors of [8] carried out research on various CNN-based algorithms, and found that MobileNet yielded the best accuracy for a masonry dataset. J. K. Chow et al. [9] carried out crack detection on concrete images using a convolutional autoencoder and decoders. Zhong qu et al. [10] and Cheng Wang et al. [11] discussed improving accuracy by using only two convolutional layers and the Inception model. SY Wang et al. [12] compared R-CNN-based ResNet, visual geometry group (VGG) and feature pyramid network (FPN). It was concluded that VGG16 took less time and memory to detect cracks, but yielding the lowest accuracy. ResNet-50 gave the highest accuracy but took more time and some extra memory. 

The addition of certain pre-processing steps can be of great value; they can make or break an algorithm. A very good example would be the research of Thendral et al. [13], where cracks on railway tracks were collected using a camera on a self-moving vehicle and various pre-processing procedurees were carried out to classify the cracks appropriately. Similarly the research by Zhong Qu et al. [10] proved that, using a simple technique of dividing an image into smaller patches, considerable improvement can be achieved, compared to most of the state-of-the-art deep learning models. CV Dung et al. [14] used a fully convolutional network (FCN) and scanned the dataset for common features on crack images and classified the images. Using the FSM module, UH Billah et al. [15] found the weak features of the dataset and eliminated them. They concatenated the encoder-decoder modules and upscaled the remaining features. This method is particularly useful when a dataset has different types of images. Although it improves the accuracy, this method is highly sensitive to the input data. 

**Figure 1:**
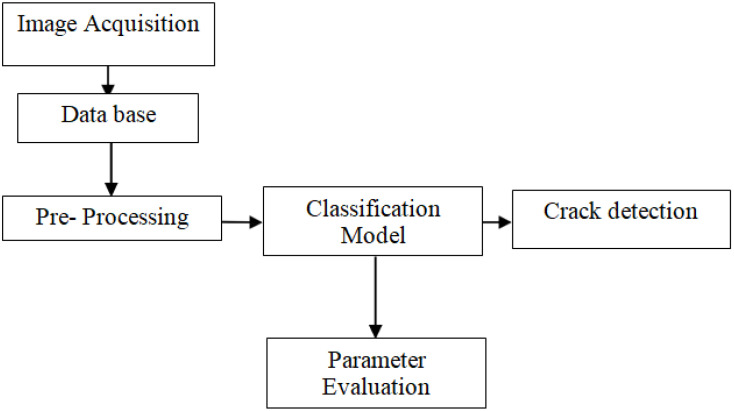
General block diagram for crack detection.

The research of Zhang et al. [16] relies heavily on the concept of feature fusion. The crack images are very susceptible to noise. It cannot be guaranteed that all the images can be taken in well-lit conditions. In similar research, the researcher used a multiscale-fusion generative adversarial network (GAN) to improve the quality of the output images while preserving the features of the original images. However, they assumed the noise type to be Gaussian and the variance to be between 0.05 and 0.2 [17,18]. Junmei Zhong et al. [19] took a different approach for reducing the noise; by using orthogonal wavelet transform (OWT), the higher scale levels are preserved, and noise in the lower level is filtered by using minimum mean squared error. Although this noise reduction method was yielding better results, they only reported it for images with Gaussian noise. Ehsan Akbari Sekehravani et al. [20], utilized the Canny algorithm for edge detection. Traditionally Canny is implemented with Gaussian filter, but to counteract any type of noise, the authors utilized a filtering approach. Another unique method is denoising the images using the Wiener filter and detecting cracks by the Otsu method [21]. Kittipat Sriwong et al. [22] and discussed various CNN-based algorithms for efficient crack detection. Even though the technique implemented in [23] was not able to carry out a proper categorization of the crack image, it could detect cracks even on road markings using ResNet-v2 algorithm. By adding feature fusion and network in network (NIN) modules, the edges were highlighted and also prevented the loss of model features, in the meanwhile reducing the time complexity. In [24], to inspect the severity of the crack, the authors used crack magnifier. The deep learning models such as VGG-16, ResNet50 and Inception ResNet-V2 are discussed in [25,26,27]. Paramanandham et al. [28] discussed about concrete crack detection using various deep learnbing models. Qi Chen et al. [29] used the guided filter approach for the removal of noise and analyzed the characterization of the crack structure using Hessian structures followed by refinement process. The authors achieved around 90% in precision, recall and F1 measurements through the implemented approach. Dawei Li et al. [30] developed a defect detection system for metro tunnel surfaces. Junjie Chen and Donghai Liu [31] proposed a model for detecting damage in the water channel based on super pixel segmentation and classification and achieved an accuracy around 91%. Miguel Carrasco et al. [32] discussed a methodology for measuring the width of cracks using smoothing, filtering, segmentation and estimation. The authors of [33,34,35,36,37,38,39,40,41] proposed several techniques based on CNN, pyramidal residual network for concrete crack detection, binocular vision system for pipe crack and deformation detection and also analyzed the performance of the techniques. From the literature, it can be identified that the existing techniques for detecting cracks can be classified into two broad domains. One is based on the combination of several networks or concentrating on segmentation of cracks. Hence, the proposed technique concentrated on overcoming the limitations in the detection of cracks even though the images are corrupted or have dissimilar structures. Figure 1 shows the general block diagram for crack detection. Once the images are acquired, the database is created. Before classifying the images into crack and non-crack, pre-processing procedures such as removal of noise, contrast enhancement, change in resolution, etc., can be performed to obtain enhanced results. Once the crack has been detected, it can be assessed through evaluation parameters.

## 3. Proposed Method

**Figure 2:**
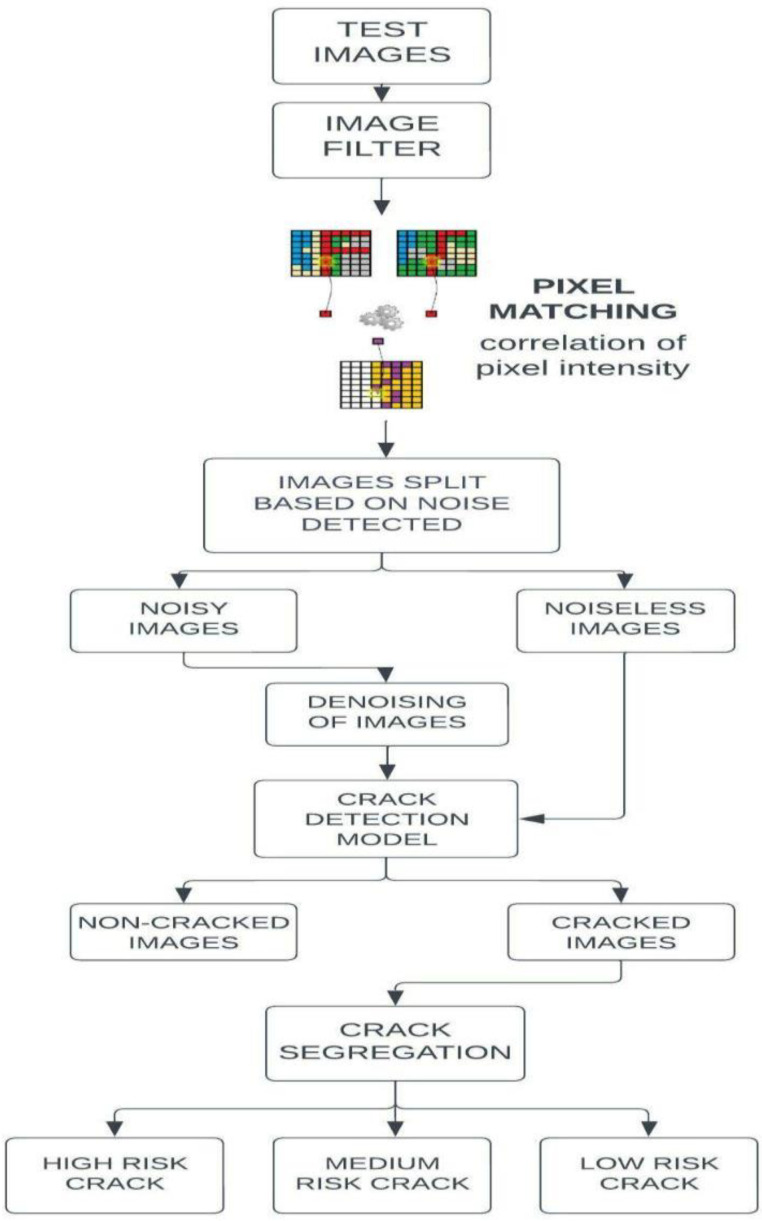
Flowchart of the proposed algorithm.

The cracks are detected for both noisy and noiseless environment images captured from various surfaces. To accomplish this, the proposed technique consists of three processes, namely, pixel-intensity resemblance measurement, crack detection using a deep learning model and classification based on the width of the crack. As shown in Figure 2, in the pre-processing stage, the filtering process and the following step (i.e.,) pixel-intensity resemblance algorithm were used for measuring similar pixels. In this pixel-matching technique, the images to be tested are passed through a common filter. The filtered image pixels are compared with the original image. The number of mismatched pixels is calculated and, according to that calculation, the type of noise is determined. Once the type of noise is identified, the proper denoising filters are used for the removal of noise. The filtered images are then segregated properly on the basis of whether the images are inclusive of noise or not. Once the separation filtering of possible noisy images is completed, the images are then passed through a crack-detection model. The images in which cracks have been detected are then passed through the last stage of the algorithm where the width of the crack is determined; by doing so, it segregates the various cracked images into three different categories based on the severity level, namely, high, medium and low so that the appropriate actions can be taken without any delay.

To examine the efficiency of the implemented technique under several noise conditions, images were generated with different noises with Gaussian, salt and pepper, and speckle with various mean and variance levels. The Gaussian noise model [37] is expressed in Equation (1).

(1) $$P(g)=\sqrt{\frac{1}{2\pi \sigma^{2}}}e^{-\frac{{\left(g-\mu \right)}^{2}}{2\sigma^{2}}}$$

where σ denotes the standard deviation, g indicates the gray value and µ represents the mean value.

The binary noise is also called impulse noise and salt and pepper, as its value is either 0 or 255. Speckle noise is also termed as multiplicative noise [37]. It occurs in the same way in an image as Gaussian noise. It is expressed in Equation (2).

(2) $$F(x)=\frac{x^{\alpha -1}e^{\frac{-x}{a}}}{(\alpha -1)!a^{\alpha }}$$

The proposed crack detection model was developed in view of the following parameters:

1. Cracks should be detected on any surfaces captured from any device under any environment;
2. Time complexity is considered;
3. Once a crack is identified, it should be categorized and an immediate alert will be given to the authority in order to avoid major accidents.

### 3.1. Filtering and Pixel-Intensity Resemblance Measurement for Noise Classification

The images of any structure or surface taken from a drone or some other device are classified into noisy and noiseless (very little noise) images, based on the measurement of pixel-intensity resemblance and a filter-based approach. To classify the images accurately, the images are initially passed through a common filter. The filtered image pixels are compared to the database images for finding similarities between the pixels. After extensive study of filters and from Table 1, it was found that the median filter yielded better results when compared to all other filters for the proposed technique. Hence, all the images were passed through the median filter and the filtered image was then given to the next stage. A median filter is a non-linear digital filtering technique that is often used in the pre-processing of images as it helps remove the noise efficiently but preserves the edge details. It is very useful for edge detection and other image-based detection methods.

**Figure 3:**
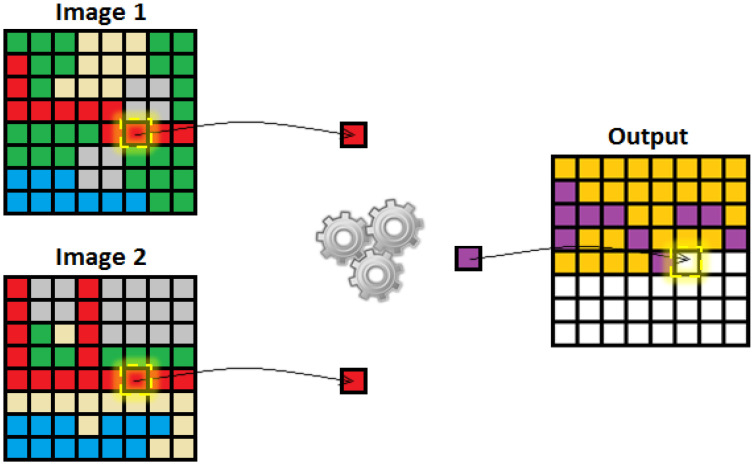
Visual representation of pixel matching process.

The filtered images are passed through the pixel-matching algorithm where the pixels of the original image and the filtered image are compared and the number of matched and mismatched pixels are computed. The code works by extracting the intensity of pixels that have the same coordinates within the image. If the pixel intensity of both images matches, then it is marked as a common pixel, otherwise it is denoted as a mismatched pixel. In Figure 3, the yellow output is the mismatched pixels while the purple output is when the pixels match.

For each image, the mismatches are compared against certain thresholds and the decision is made whether to use the original image or the filtered image. If the number of mismatched pixels is between 15 and 100 then the original image is passed through to the deep learning model, as the filtering of images with supposedly very little or no noise ends in needless loss of the image. The images whose pixel mismatch range is over 100 are deemed to be noisy, the original image is first filtered and then passed through the detection algorithm.

### 3.2. Noise Estimation

In order to decide the filter that should be used for denoising, the level of noise is estimated and the flow for the estimation is explained using the Equations (3)–(8). Noise is estimated for various types of noises with different mean and variance levels. Let us consider an image I with the patch size p, row R and column C and dataset D that is specified in Equations (3) and (4)

(3) $$I\;\epsilon {\;A}^{\mathrm{RXCX}3},\;$$

and

(4) $$D={\left\{d_{i}\right\}}_{i=1}^{s}$$

where D contains s = (R − d + 1) (C − d + 1) patches with size q = 3p^2^

(5) $$\mu =\overset{s}{\underset{i=1}{\sum }}x_{i},$$

(6) $$\mathrm{Covariance}\;\mathrm{Matrix}\;\sum =\frac{1}{s}\overset{s}{\underset{i=1}{\sum }}(x_{i}-\mu ){\left(x_{i}-\mu \right)}^{t}\;.{\;}^{\;}$$

Computing the Eigen values ${\left\{\lambda_{i}\right\}}_{j=1}^{q}$ of the covariance matrix Σ with q = p^2^ and order λ_1_ ≥ λ_2_ ≥ … ≥ λ_r_

For j = 1: q, median τ is calculated

(7) $$\tau =\frac{1}{q-t+1}\overset{q}{\underset{k=1}{\sum }}\lambda_{k}\;,$$

If τ is the median of the set ${\left\{\lambda_{k}\right\}}_{k=1}^{q}$ then,

(8) $$\sigma =\sqrt{\tau }\;.$$

where σ represents the estimated noise level.

**Figure 4:**
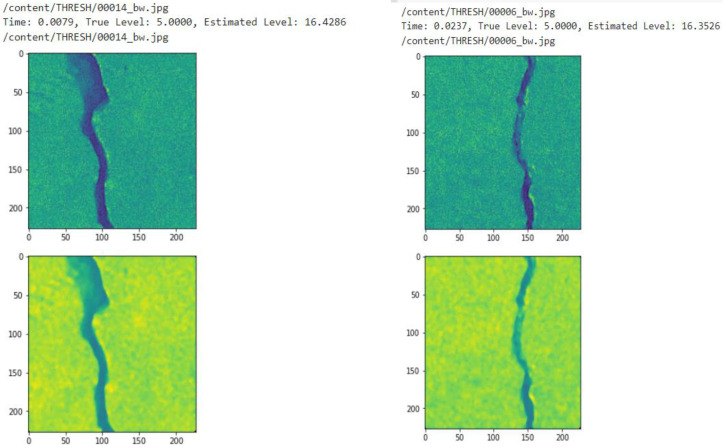

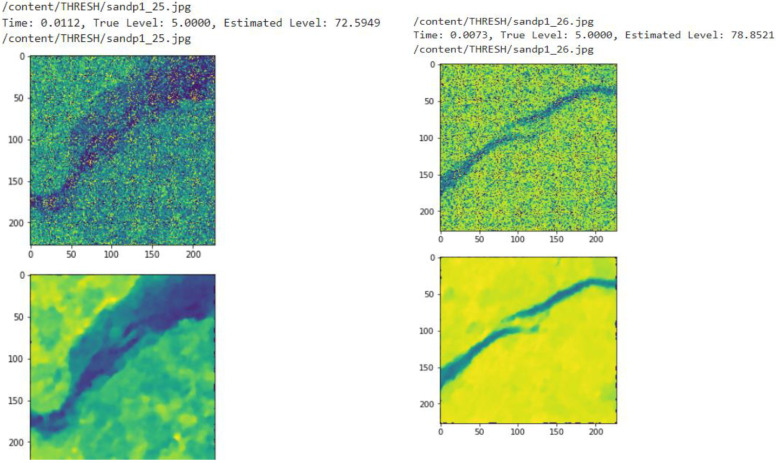
Noise estimation.

If the estimated range is within ±5, it is lying under Gaussian noise and the Wiener filter is chosen for denoising these types of images as it is more appropriate. Similarly, an image with the estimated range 15 to 25 specifies speckle noise, the mean filter is used for denoising and if it is greater than 30, it shows the image is corrupted due to salt and pepper. If the images are corrupted by salt and pepper noise, the median filter is used for the removal of noise. Figure 4 shows the noise estimation of the proposed technique.

### 3.3. Deep Learning Models Used for Crack Detection

#### 3.3.1. VGG-16 Architecture

**Figure 5:**
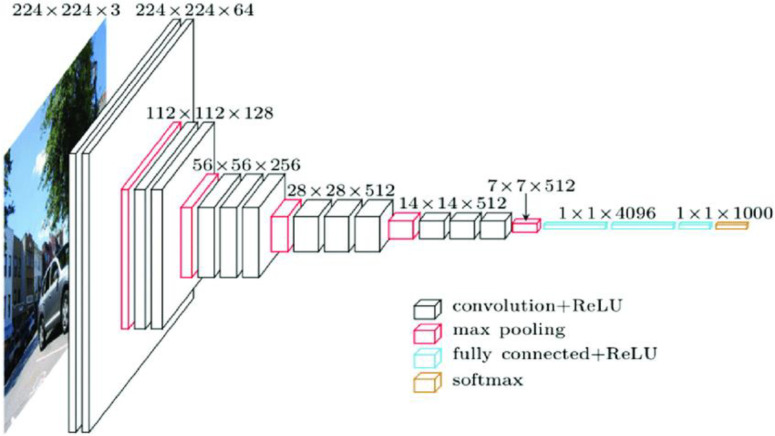
Architecture of VGG-16.

The VGG-16 consists of 13 convolutional layers and three fully connected layers as shown in Figure 5. A set of filters comprises a convolutional layer which is an essential block in any convolutional neural network. VGG-16 [25] has 13 of them. The parameters of the filters have to be learned. The size of the filter must be relatively less than the input. The features of the training set are extracted only using convolutional layers. The next layer in VGG-16 is the pooling layer. Generally, pooling layers are added between two convolutional layers. Pooling layers reduce the number of parameters between successive layers. There are two pooling functions, namely average and max pooling. Max pooling is generally preferred as it functions more efficiently. The flattened layer in VGG-16 converts feature maps into 1D tensors. The last layer is the fully connected layer which gives the output of the model.

#### 3.3.2. ResNet-50 Architecture 

**Figure 6:**
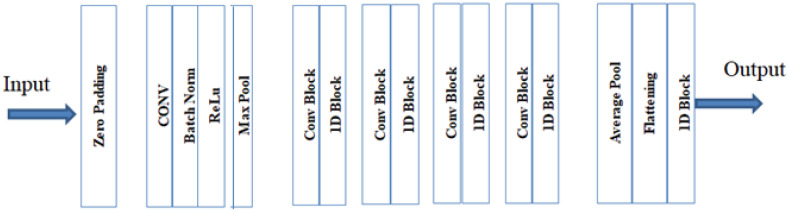
The architecture of ResNet-50.

ResNet-50 is a variant of ResNet with 50 neural network layers [26] as shown in Figure 6, redrawn from [39]. Over the years, the higher accuracy and efficiency of neural network models have been achieved by deepening the neural network model, i.e., adding more layers and blocks or changing the filter size. This, however, is not always the case. Adding more and more layers can also cause performance degradation in deep learning. In order to overcome this, residual networks which are made up of residual blocks have been invented. The concept of skip connection is being introduced in residual models. While training a model, the skip connections skip some of the layers in the model (layers that are skipped vary from model to model). The output of one layer is fed as the input to another layer. This basically solves the problem of vanishing gradients in deep neural networks. The skip connections also ensure that the higher layers and lower layers of a model perform efficiently. The residual blocks in the model help to increase efficiency as learning becomes much easier.

#### 3.3.3. Inception ResNet-V2

**Figure 7:**
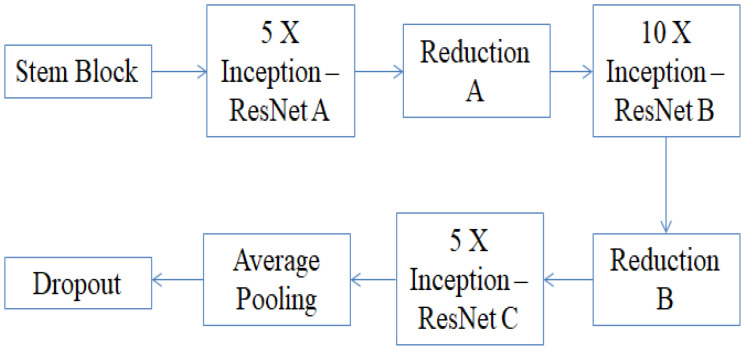
The architecture of Inception ResNet-v2.

Inception ResNet-v2 basically uses the inception architecture combined with the residual connections from the ResNet network. The major improvement from the traditional model is the addition of a filter expansion layer to scale up the dimensionality of the filter bank before the addition to match the depth of input. The network has a total of 164 layers, as shown in Figure 7, redrawn from [39], and can classify the images in up to 1000 different categories, in the same way as the VGG-16 and ResNet-50. The input size of this network is 299 × 299 and the output is a list of estimated class probabilities.

#### 3.3.4. Xception Model

**Figure 8:**
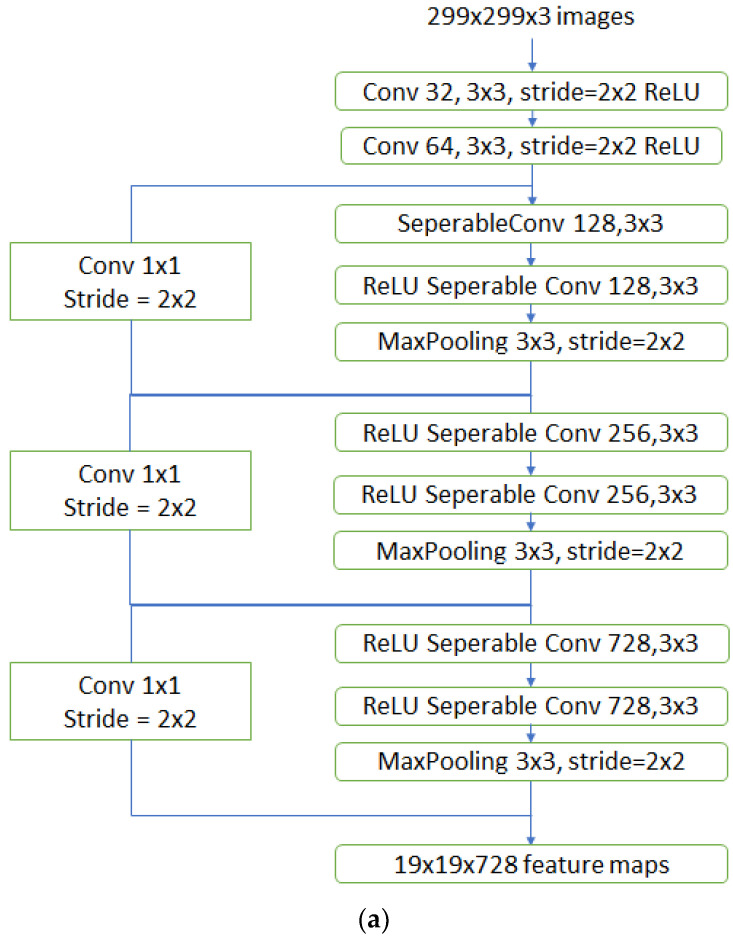

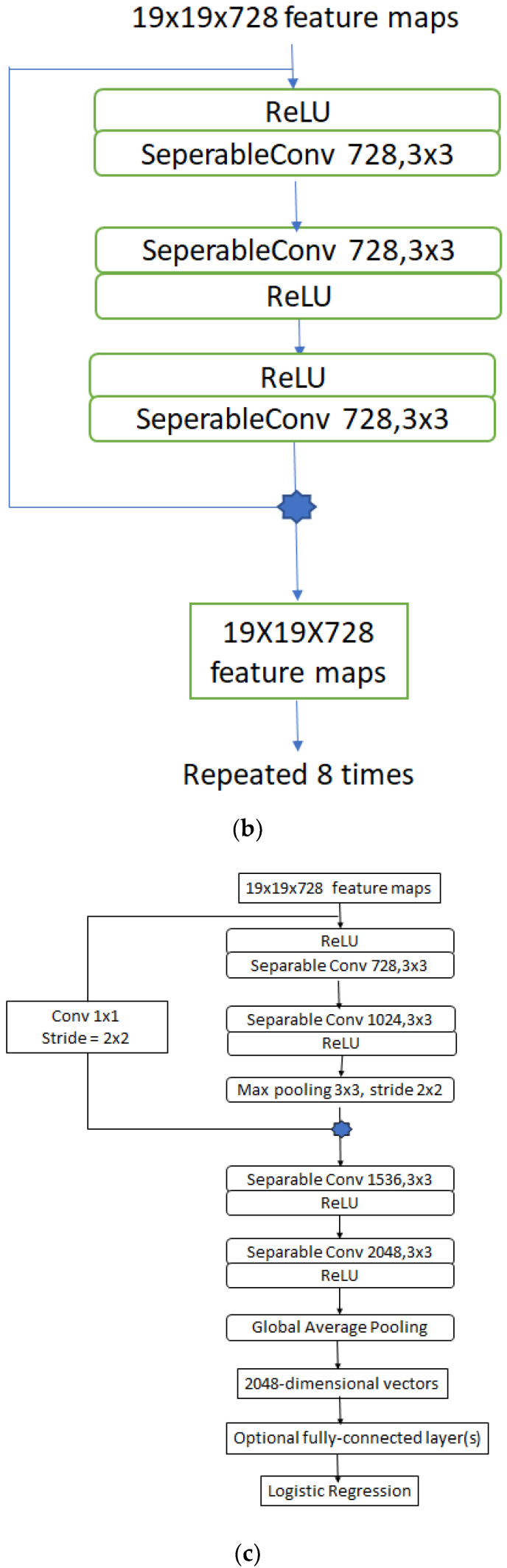
(**a**) The architecture of Xception entry flow; (**b**) the architecture of Xception middle flow; (**c**) the architecture of Xception exit flow.

The Xception model uses depth-wise separable convolutions and works as shown in Figure 8a–c, redrawn from [27]. A general convolution step makes the spatial-wise and channel-wise computation in one single step. However, on the other hand, the depth-wise separable convolution divides the process of computation into two different steps. The depth-wise convolution initially adds a single convolutional filter to each input channel. It is then followed by point-wise convolution which creates a linear combination of the output from the depth-wise convolution. This method improves the efficiency of the model.

The word “Xception” literally translates as “extreme inception”. It basically means that the properties of the inception model are extremized to give better results. In the traditional inception neural network model, the original input image was compressed using a one-by-one convolution. After this, different types of filters were used on each depth space. However, in the Xception model, this step is reversed. Here, the filter is applied in the first step of the depth map and then the compression of the input takes place. This technique is called depth-wise convolution. The Xception model also does not introduce non-linearity which was the case in the inception model. This is also yet another difference between the models.

### 3.4. Crack Segregation Based on BSE Algorithm

The images that are identified with cracks are passed to the proposed crack risk analysis algorithm (binarization-skeletonization-edge detection—BSE) where the width of the crack is estimated. Based on the width, the images are segregated into high-risk, medium-risk and low-risk cracks by the preset threshold. 

Crack risk analysis using BSE algorithm:

1. Image binarization: this is the operation of dividing the image into black/white pixels in order to separate the cracks and non-cracks within the image;
2. Skeletonization: extracts the central skeleton of the crack which helps to identify the progression of the crack. Hence, it is possible to find the crack width by drawing a line perpendicular to the crack propagation direction at the pixel on the skeleton;
3. Edge detection: extracts the outline of the crack. From the skeleton, the line perpendicular to the crack propagation direction and the crack outline are used together to find the crack width.

## 4. Results and Discussion

**Figure 9:**
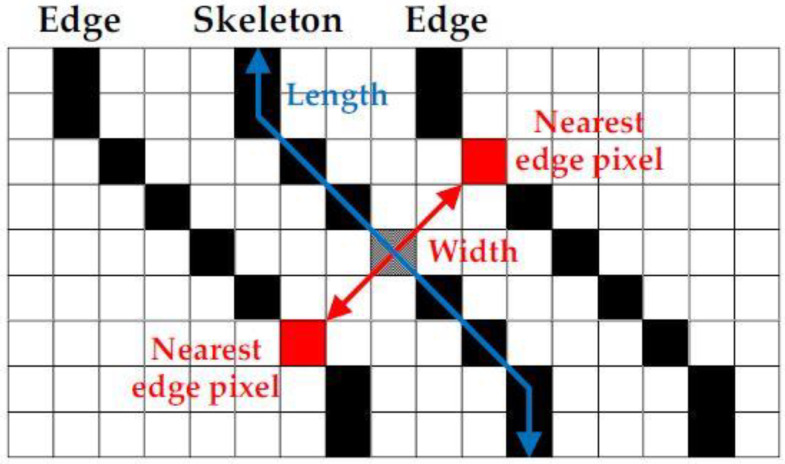
Visual representation of predicting the crack width.

**Figure 10:**
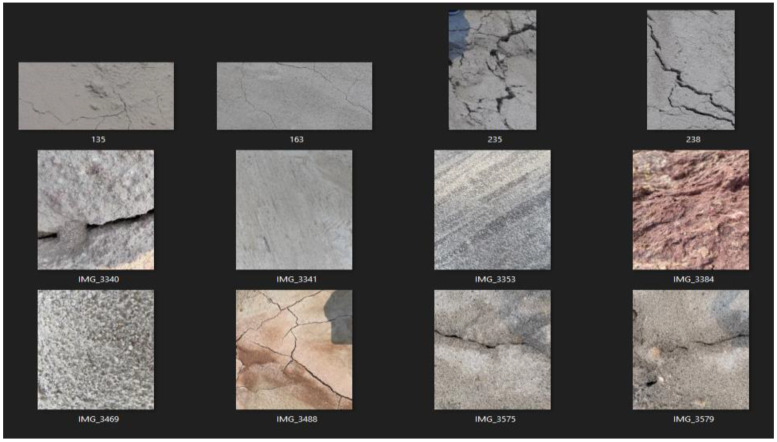
Real-time sample crack images used in the experiment.

**Figure 11:**
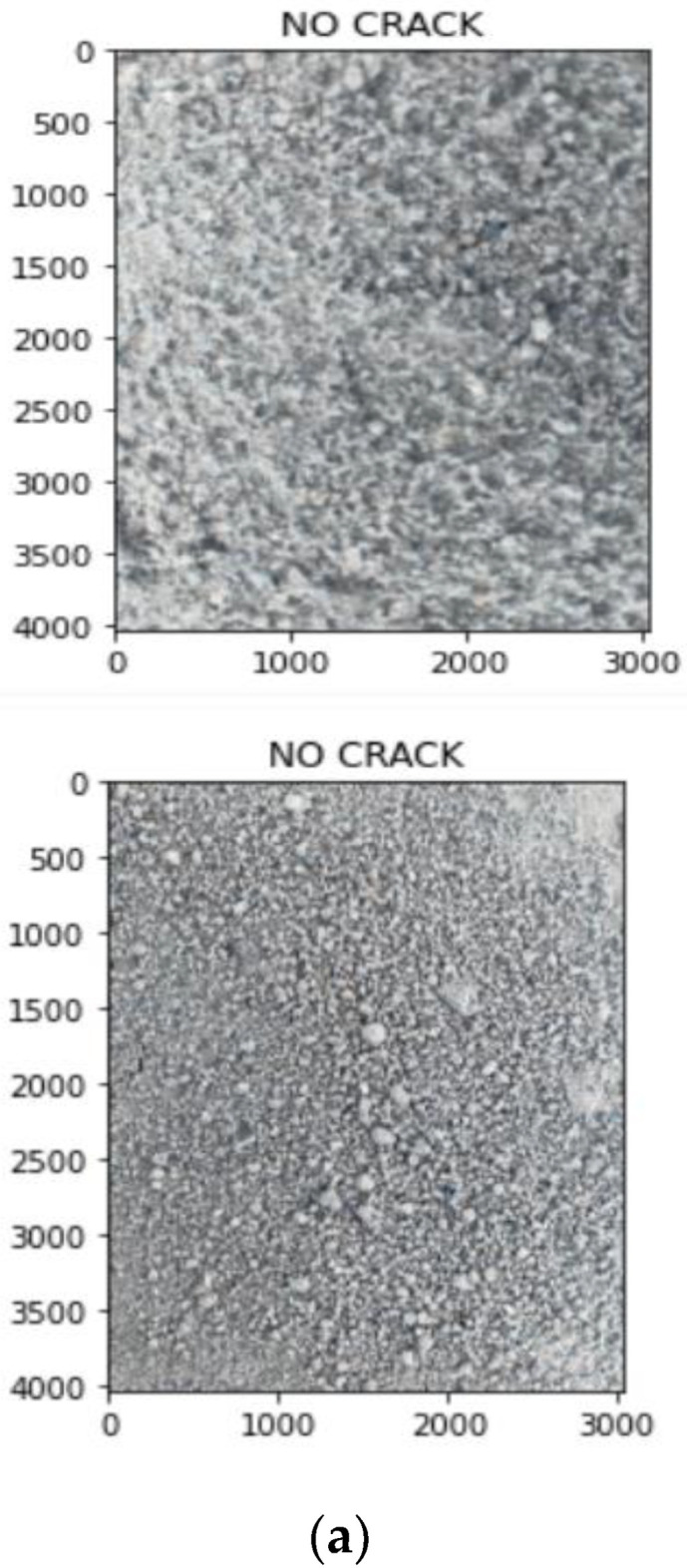

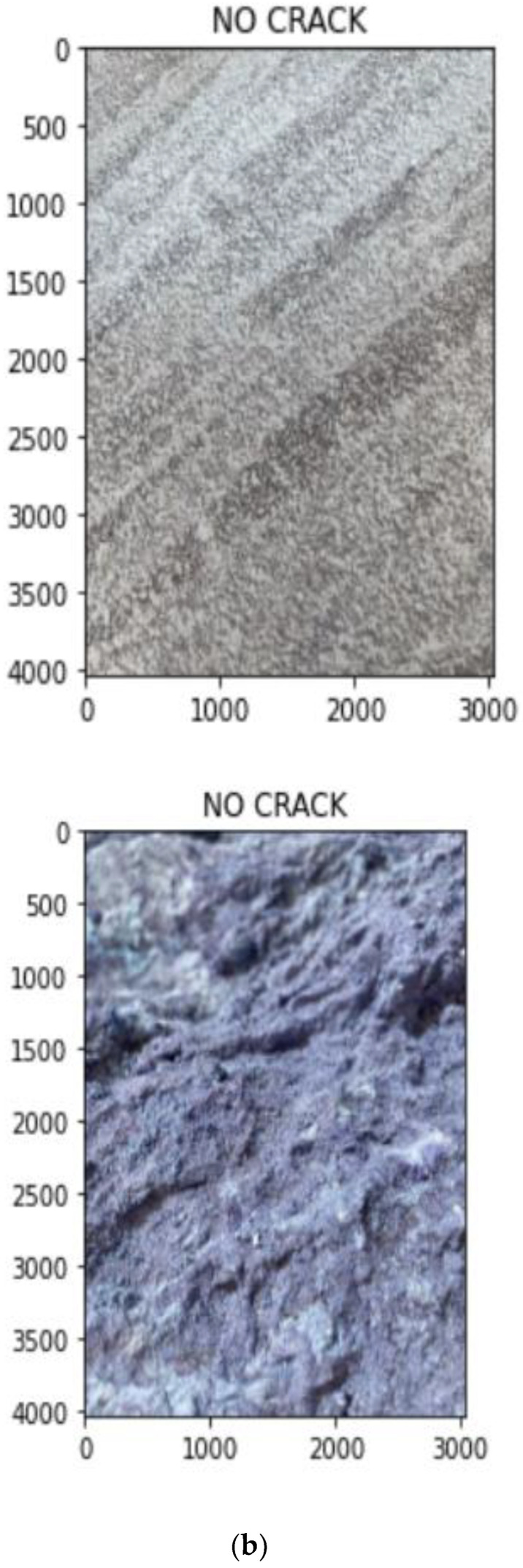
(**a**,**b**) Sample output images from the model classified as “No Crack”.

**Figure 12:**
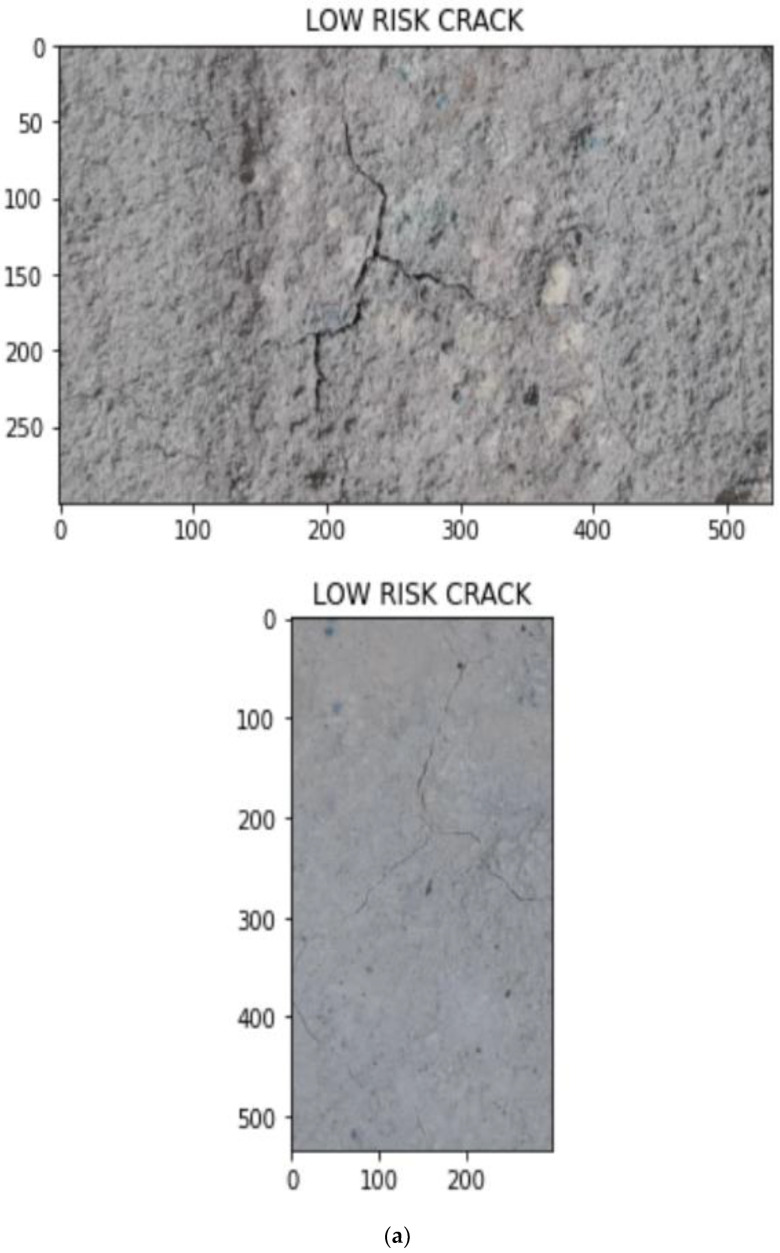

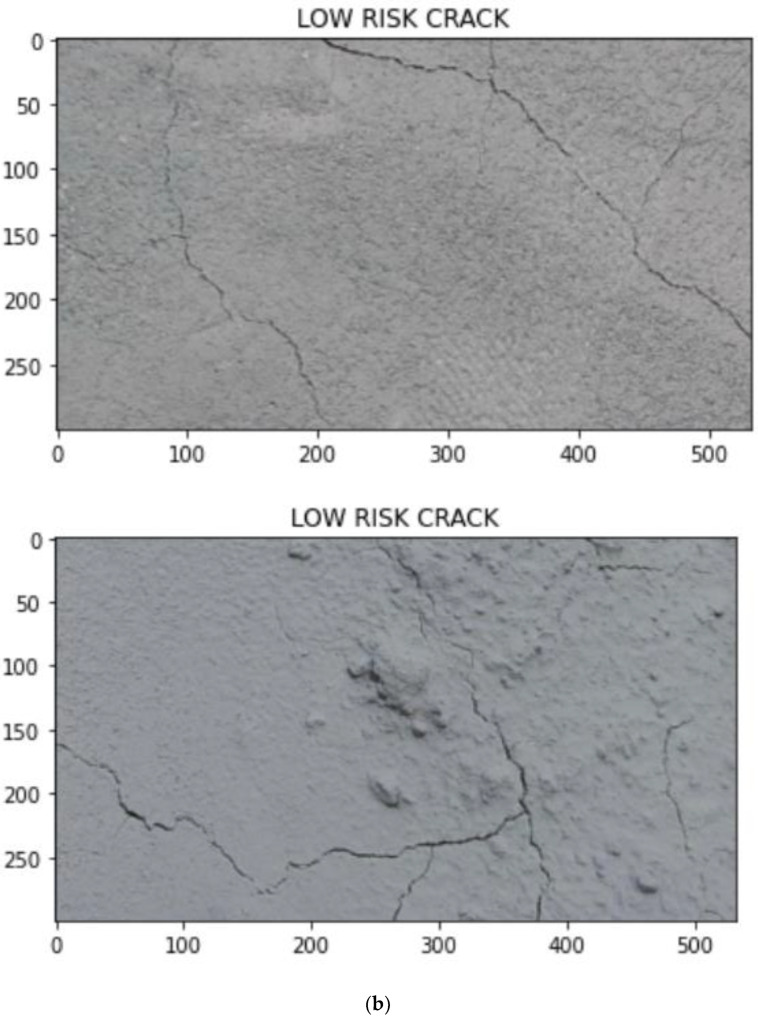
(**a**,**b**) Sample output images from the model classified as “Low-Risk Crack” for field images.

**Figure 13:**
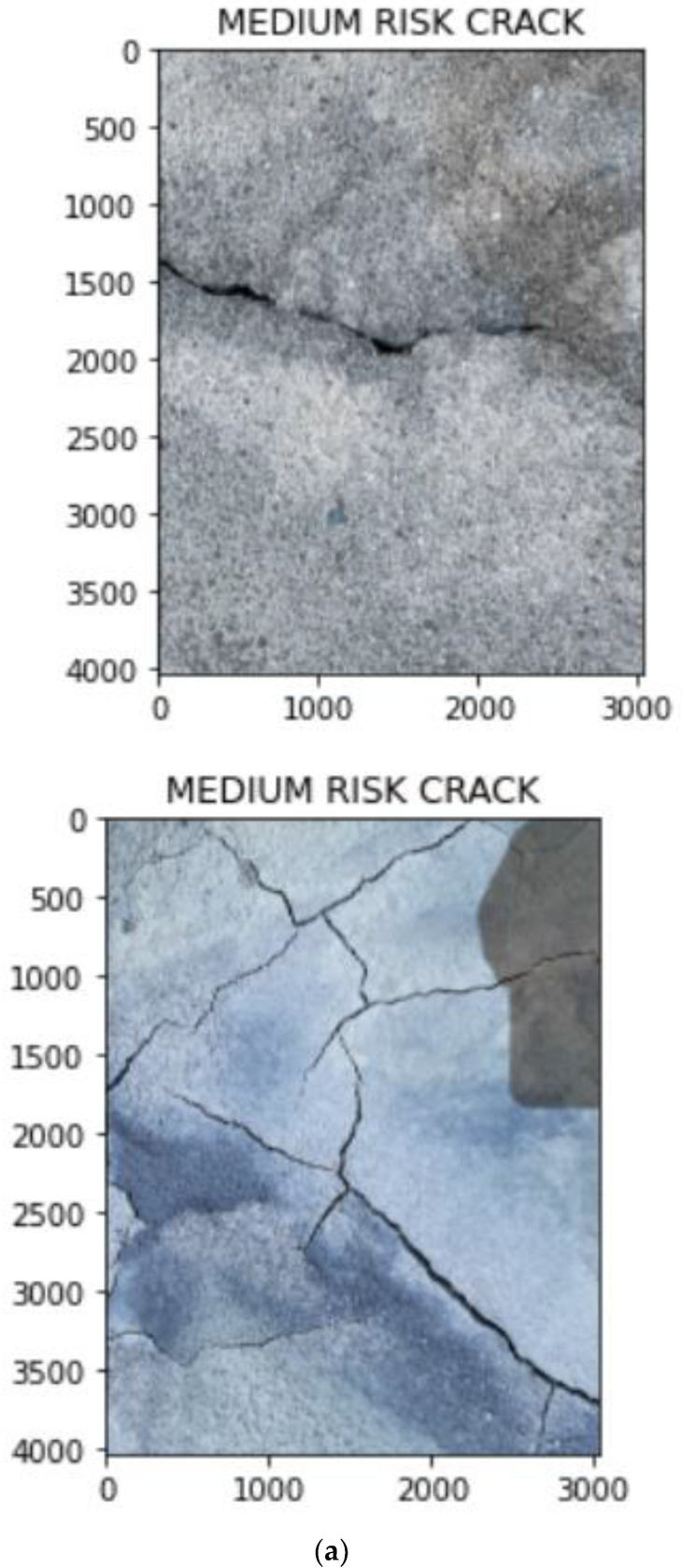

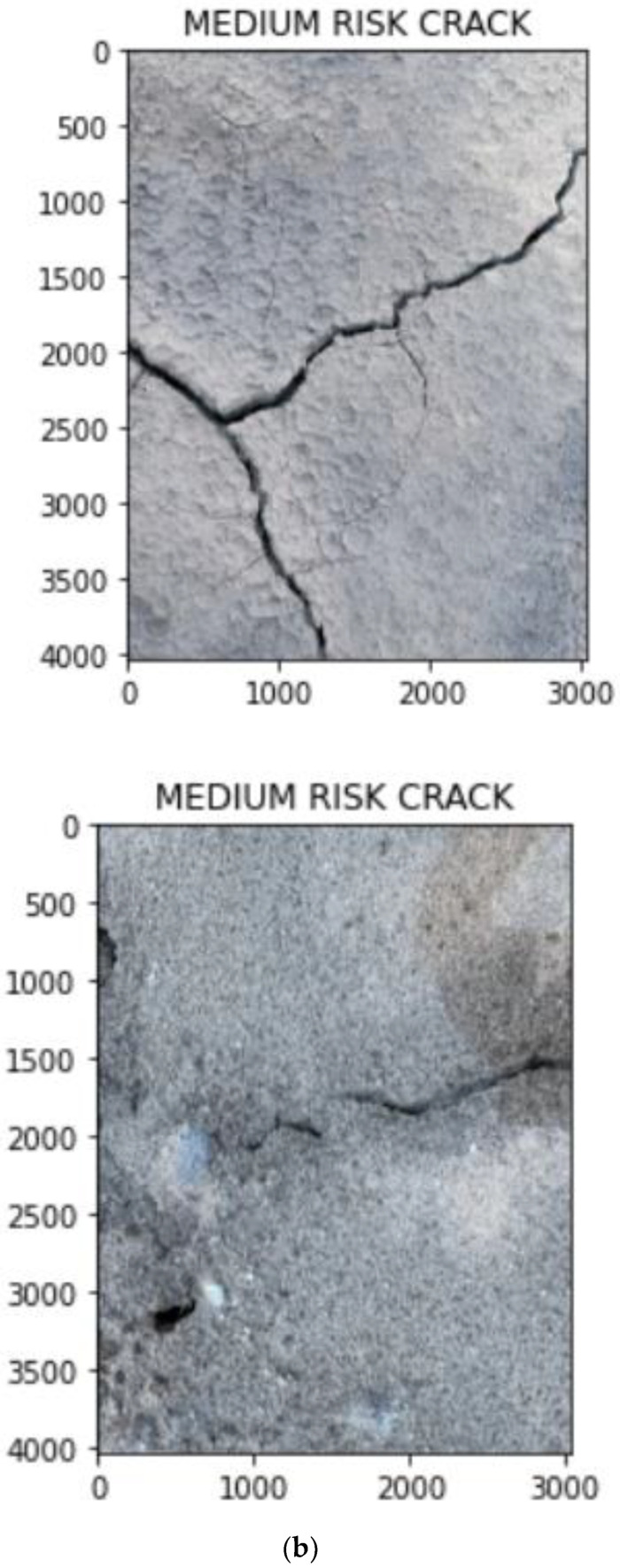
(**a,b**) Sample output images from the model classified as “Medium-Risk Crack”.

**Figure 14:**
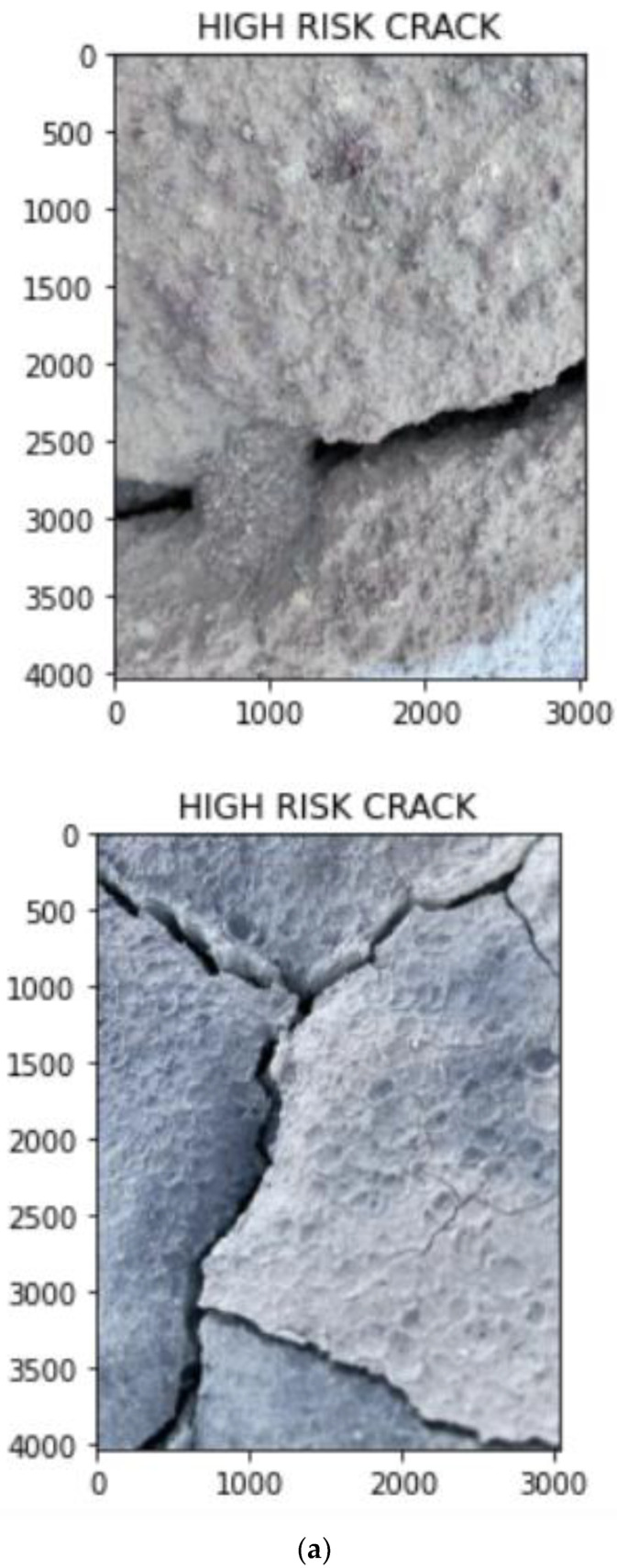

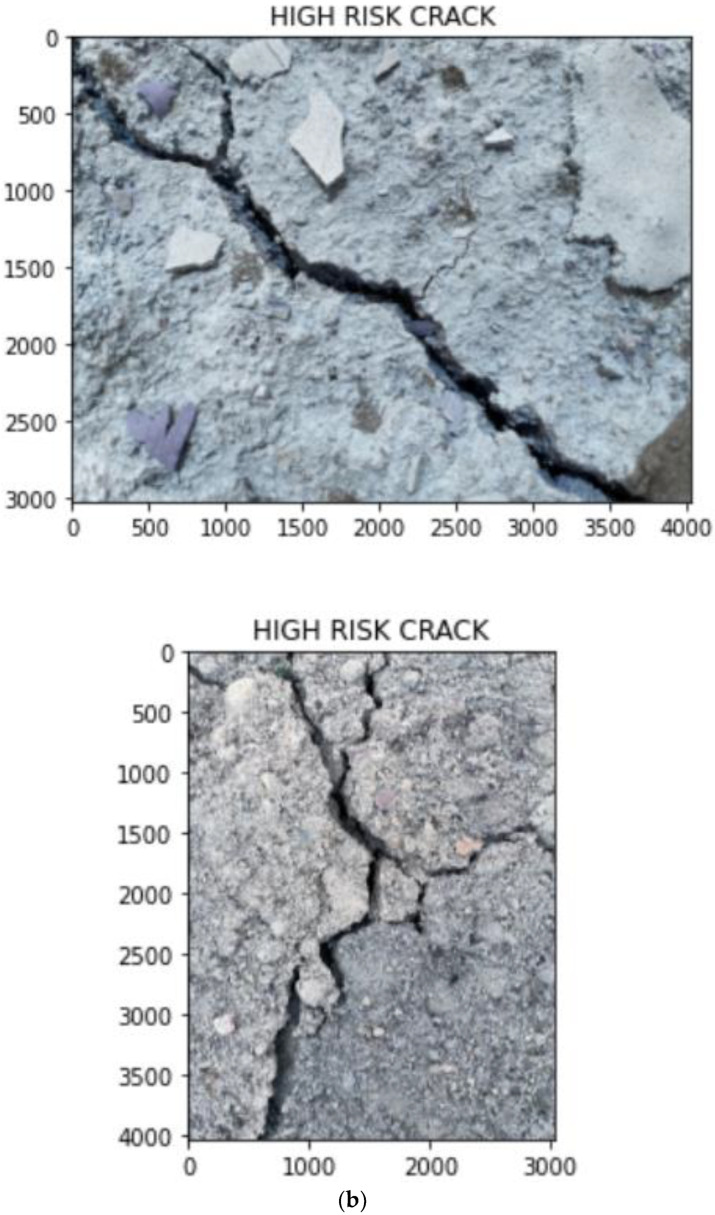
(**a,b**) Sample output images from the model classified as “High-Risk Crack”.

Once the type of noise is estimated, the appropriate filters are applied. These images are converted into gray scale images and the width is calculated. The crack estimation accuracy is drastically increased when the images are denoised based on the proposed technique. For calculating performance evaluation parameters, confusion matrix is used and it is represented in Table 2. Table 3 and Table 4 show the accuracy of various deep learning models before and after denoising. From Table 3 and Table 4, it is proved that the proposed technique is efficient in denoising and detecting the cracks. Figure 9 shows the visual representation of crack width prediction. Once the crack was identified, it was classified into high risk, medium risk and low risk and these are shown in Figure 10, Figure 11, Figure 12, Figure 13 and Figure 14. The high-risk cracks would be immediately alerted to the authority to ensure avoidance of major disasters or accidents and to prevent a calamity. The work was implemented using Python software in Google Colab. The proposed system will be very helpful to many industries and public transport authorities, including bridges on the pathway. To assess the effectiveness of the proposed technique, it was compared with state-of-the-art techniques such as Auto-CAE [9], ResNet-50 [26], Crack Hessian [29] and Seg + SVM [30] and the results are tabulated in Table 5.

By searching the skeleton of the image through breadth-first search (BFS), the direction of the crack was estimated. Then the distance was calculated when the line of perpendicular met the edge of the crack, as shown in Figure 9. This was repeated multiple times until various widths were covered and the average of the distances obtained was used as the estimated width value of the crack. Figure 10 shows some sample images collected from industry. With multiple hand-selected images of various degrees of severity in the crack, accurate thresholds for high, medium, and low risk were identified by the proposed model. Figure 11a,b shows the sample of predicted output of the model “No Crack”, even though the images have many irregularities, grainy surface and complicated structure. Figure 12a,b shows the predicted output of the model “Low-Risk Crack”, Figure 13a,b shows the classified output of “Medium-Risk Crack”. Once a high-risk crack is detected, an immediate alert will be given to the authority and the necessary action will be taken to avoid accidents. The high-risk-classified crack images are shown in Figure 14a,b. Even though the images have different surface properties, the proposed model can effectively classify according to category. A confusion matrix was generated for all the models to assess the efficiency of the models and this is given in Table 2.

This table indicates the predictions made by the model and how right/wrong those predictions were. The parameters such as accuracy, precision, recall and F1 score were calculated using Equations (9)–(12), respectively.

(9) $$\mathrm{Precision}=\frac{\mathrm{TP}}{\mathrm{TP}+\mathrm{FP}},$$

(10) $$\mathrm{Recall}=\frac{\mathrm{TP}}{\mathrm{TP}+\mathrm{FN}},$$

(11) $$F1\;\mathrm{Score}:\;2\;X\;\frac{\mathrm{Precision}\;X\;\mathrm{Recall}}{\mathrm{Precision}+\mathrm{Recall}},$$

and

(12) $$\mathrm{Accuracy}:\;\frac{\mathrm{TP}+\mathrm{TN}}{\mathrm{TP}+\mathrm{TN}+\mathrm{FN}+\mathrm{FP}}.$$

## 5. Discussion and Conclusions

The adverse effects of noise on image-based detection methodologies, especially in the detection of cracks, are successfully identified in this article by exploring various deep learning algorithms. The discussed deep learning models showed around 30–50% decrease in accuracy when the test images were noisy. To counteract this, the noise was estimated and the appropriate filters were used for denoising using the developed technique. From the results, it was identified that the implemented technique had different effects on the various models. When the dataset contained all the types of images excluding the images corrupted from Gaussian noise, speckle noise, Poisson noise, salt and pepper noise, the proposed technique achieved 6% improvement without PIRM and 10% improvement with the PIRM rule for the VGG-16 model. Similarly, it showed a 3 and 10% improvement for ResNet-50, a 2 and 3% improvement for Inception ResNet and a 9 and 10% improvement for the Xception model. When the images were corrupted from single noise, 95.6% accuracy was achieved using the ResNet-50 model for Gaussian noise, 99.65% accuracy was achieved through Inception ResNet-v2 for Poisson noise, and 99.95% accuracy was achieved by the Xception model for speckle noise.

From these results, it was concluded that the ResNet-50 model was the most suitable both when the test images contained no noise, as well as for all types of noisy images, achieving 95.78% with the proposed technique. To evaluate the performance of the developed technique, it was compared with state-of-the-art techniques and the obtained results depicted that the proposed technique outperformed the existing techniques. Thus, it can be concluded that the pixel-intensity resemblance measurement, noise estimation and crack classification-based technique proposed here is most suitable for all types of real-time images taken from any environment. 

In future, the authors plan to work on the limitations of the proposed work, i.e., detecting cracks and uneven surfaces occurring in various materials such as steel, iron, and compound cylindrical structures due to strain or some other external environmental factors.

## Figures and Tables

**Table 1:** Noise estimation through filters with PIRM.

Image Filters	Classification Accuracy (%)
Mean Filter	87.3
Median Filter	91.5
Low Pass Filter	83.3
Gaussian Filter	88.1

**Table 2:** Confusion matrix.

	Predicted No	Predicted Yes
Actual No	TN	FP
Actual Yes	FN	TP

**Table 3:** Accuracy with and without noise.

NOISE	VGG-16	RESNET-50	INCEPTION RESNET-V2	XCEPTION
No noise	99.9%	98%	99.98%	99.95%
Salt and pepper	50%	50%	56.05%	50%
Gaussian	50%	89.05%	56.15%	50%
Poisson	99.1%	99.2%	98.75%	99.25%
Speckle	96.05%	91.9%	99.55%	99.7%
All noises	73.79%	82.54%	85.47%	74.74%
All noises + no noise	79.41%	85.63%	88.36%	79.79%

**Table 4:** Accuracy of various models using proposed technique.

NOISE	VGG-16	RESNET-50	INCEPTION RESNET-V2	XCEPTION
Salt and pepper	81%	88.7%	96.3%	99.65%
Gaussian	50%	95.6%	90.95%	50%
Poisson	99.1%	99.2%	99.65%	99.25%
Speckle	99.2%	98.2%	99.85%	99.95%
All noises	82.25%	86.25%	87.89%	87.23%
All noises + no noise	85.79%	88.32%	90.3%	88.74%
All noises + no noise (**with PIRM**)	89.56%	95.78%	90.9%	89.57%

**Table 5:** Comparison with the state-of-the-art techniques.

Techniques	Accuracy	Specificity	Recall	Precision	F1 Score
ResNet-50 [26]	88.36	89.06	87.16	89.46	88.11
Auto-CAE [9]	89.05	89.95	87.32	90.05	88.75
Crack Hessian [29]	91.2	91.9	90.03	91.72	90.9
Seg+ SVM [31]	91.7	91.05	90.27	91.35	91.23
**Proposed- (PIRM + BSE)**	**95.78**	**96.48**	**94.38**	**96.18**	**95.58**

## Data Availability

Publicly available data is used and cited appropriately.
